# The Hospital. Nursing Section

**Published:** 1905-02-25

**Authors:** 


### The Hospital.

### Nursing Section.

Contributions for this Section of "THE HOSPITAL" should be addressed to the EDITOR, "THE HOSPITAL," NURSING SECTION, 28 & 29 Southampton Street, Strand, London, W.C.

No. 961.—VOL. XXXVII. *SATURDAY, FEBRUARY* 25, 1905.

### Notes on News from the Nursing World.

### THE NEW MATRON OF THE ROYAL FREE HOSPITAL.

THE election of the new matron of the Royal Free Hospital, Gray's Inn Road, took place on Wednesday afternoon. There were nearly fifty candidates from all parts of the United Kingdom and Ireland. The final choice of the Governors fell upon Miss Cox Davies, at present matron of the New Hospital for Women in Euston Road. Miss Cox Davies was trained for three years and was awarded the gold medal at St. Bartholomew's Hospital, where she was afterwards night superintendent, home sister, housekeeper, and matron's secretary. During part of this period, 1896 to 1902, she served in South Africa as a member of the Army Nursing Service Reserve. In May 1902 she was appointed matron of the Devon and County Hospital, Exeter, but resigned in October of that year in consequence of being elected matron of the New Hospital for Women, a position which now becomes vacant. In view of the ripe and varied experience of Miss Cox Davies, and the special knowledge acquired in her last post, which should be specially valuable in the administration of affairs at the Royal Free Hospital, we think that a very suitable successor to Miss Wedgwood has been selected.

### ROYAL NATIONAL PENSION FUND FOR NURSES.

THE annual general meeting of the members of the Royal National Pension Fund for Nurses will be held on Thursday, March 9, at River Plate House, Finsbury Circus, E.C., at 4 P.M. It is the hope of the Council that a large number of policy-holders will be present. Those who accept the invitation have merely to write their names and policy number legibly on a card to be handed in upon entering the room. The proceedings at the annual meetings are always full of interest, and the continued growth of the Fund will no doubt furnish excellent material for the speakers on the 9th of next month.

### THE QUEEN OF ANOMALY.

AT the meeting convened by the Matrons' Council at Hanover Square, on Wednesday, a letter was read from Miss Peter in favour of registration; she urged that a protest should be lodged with the Board of Trade against the Society for Promoting the Higher Education of Nurses. By a curious coincidence we have received a letter this week from the United States, informing us that Miss Peter is going to Massachusetts to support the scheme now being organised there by Dr. Worcester, which is denounced by the advocates of State registration in the United States, on grounds mainly identical with those upon which the Matrons' Council are opposing Lord Rothschild's scheme. The nursing world in England will, no doubt, be glad to learn how Miss Peter justifies this twofold attitude, which entitles her to be considered for the moment the Queen of Anomaly.

### THE NURSING OF TYPHUS FEVER.

ALTHOUGH there is reason to hope that the outbreak of typhus fever which occurred in the East End of London last week may not attain any serious dimensions, we think that the contribution which appears in another column, of a nurse who was on duty through the typhus epidemic in Lancashire a few years ago, will be read with special interest just now. As will be seen from her article, there were nearly 200 cases in a month, though the percentage of deaths was low. The superintendent of the Homerton Fever Hospital attributes the outbreak at Mile End to the use of second-hand clothes tainted with the disease. There is no doubt that the most important factors favouring the spread of typhus fever are dirt and defective sanitation in the homes of the people. It is owing to the great improvements which have been made during recent years in domestic hygiene that typhus fever has become a comparatively rare disease.

### DISTRICT NURSING AND MIDWIFERY.

THE energy with which district nursing is being carried on in the urban and rural districts of Devon is attested by the generally satisfactory reports just now being presented at the annual meetings. At Exmouth the nurse made 6,587 visits in 1904 (against 5,474 in 1903) to 141 medical and 90 surgical cases; at Teignmouth the nurse attended 111 cases, making 3,420 visits; at Penryn, the record was 4,349 visits to 127 cases; at Callington 88 cases, making 2,396 visits, besides 14 maternity cases; at Bideford 3,578 visits to 112 cases; at South Brent 1,630 visits; at St. Germans, two nurses made 2,470 visits to 84 cases; at St. Ives two nurses paid 3,260 visits in 127 cases, while there were 25 maternity cases; at Ashburton 2,259 visits were made in 50 cases; at Crediton 3,876 visits were paid and 28 maternity cases attended by two nurses, and at Totnes the record was 3,281 visits to 99 serious cases. Most of these organisations are now adding midwifery to the district nurses' duties. This circumstance, and the fact that many other district nursing associations in the county are combining midwifery with district nursing, renders it necessary for us to point out that the policy is one which involves serious risks. In the rules passed by the Central Midwives Board, in pursuance of Section 3 of the Midwives Act, there is one, Rule E 15, which bears upon the subject of the combination of the offices of midwife and district nurse. It runs as follows: "No midwife shall undertake the duty of laying out the dead, or follow any occupation that is in its nature liable to be a source of infection." The Board, it is therefore clear, discourages the union of the two offices; but the primary responsibility for carrying out the rule rests, of course, with the local supervising authority in each case. It is obvious that district nurses in the performance of their ordinary duties must frequently come into personal contact with septic and other infectious material. Under the arrangements which exist at the places we have mentioned above, they will almost certainly convey infection sooner or later to some poor mother, with terrible results. This is a peril which it is absolutely essential to avoid, or the state of poor mothers and their infants may be worse under the Act than it was before it was passed.

### THE LOCAL GOVERNMENT BOARD AND PLYMOUTH GUARDIANS.

As we anticipated, the Local Government Board are not likely to accede to the request of Plymouth Guardians to dismiss the superintendent nurse without due inquiry. They have been informed by Miss Holliday that neither the guardians nor the medical officer have made any complaints as to her ability, and also that when in June 1902 the guardians investigated the resignation of nurses they passed a resolution in which they wished to express their confidence in the management of the superintendent nurse, while last year, when the guardians experienced difficulty in procuring nurses, the hospital committee passed a resolution that no official was to blame in the matter. Miss Holliday has carried the war into the enemy's camp by suggesting an inquiry into the whole question of the management of this workhouse infirmary. She avers that there has been considerable difficulty in the management during the past year, which makes it almost impossible for the superintendent nurse to carry out the regulations of the Local Government Board or the medical officer's instructions. The outcome of her letter is that the guardians have been requested to furnish the Local Government Board with—what Miss Holliday has not received—a copy of the precise charges against the superintendent nurse, a statement of the witnesses heard, and the evidence taken in regard to them. The letter has been referred to the hospital committee, and their report is to be considered by the board *in camera*.

### PENSIONS FOR IPSWICH NURSES.

At a conference held last week at the Ipswich Nurses' Home, the question of private nursing was discussed, and Major Meller, the Vice-President, said he did not think that the earnings of the private nurses should be taken to pay for the nursing of the poor of Ipswich. He would like to see the profit from that department set aside for the nurses themselves, so as to secure them a pension when they were no longer able to work. As it has always been our contention that the earnings of private nurses should not be taken to pay for the nursing of the sick poor, we are glad that Major Meller is prepared to exert his influence to prevent the perpetuation of a vicious practice, and in favour of the sound and just principle that private nurses should themselves derive the benefit of any profit arising from their earnings. No better plan than that of hall-marking it for securing them pensions through affiliation with the Royal National Pension Fund for Nurses could be proposed, and we hope to hear shortly that the admirable arrangement has been adopted.

### CONTRIBUTIONS OF GUARDIANS TO NURSING ASSOCIATIONS.

The Chesterton Guardians have sanctioned, "as a temporary measure," a contribution of £25 per annum to the Cambridge District Nursing Association towards the salary of a nurse at Chesterton on condition that assistance be given to out-door relief cases. It will be recollected that the Finance Committee advised the Guardians to offer to contribute a sum equal to a half-penny rate towards the support of a fully-qualified nurse in each medical district, with the understanding that the voluntary subscriptions raised in the district should suffice to provide the balance needed for the nurse's salary. This would have meant £46 a year in each instance, and we are sorry that the recommendation was withdrawn. The result of the contribution of £25 which has been voted in one case only will not, we think, be satisfactory either to the Guardians or to the association.

### THE STANDARD OF EDUCATION FOR MIDWIVES.

At the annual meeting of the Shropshire Nursing Federation, Mrs. Baldwyn Childe, who was in the chair, reminded the members that Shropshire was the first county in England, 10 years ago, to start the training of midwives with county council money; and she mentioned that several of the nurses who were trained then were still working in the county. On this question, Miss Davies, superintendent of the East Ham Nurses' Home, a branch of the Plaistow Home, who read a paper at the meeting, said that there now prevails a strong impression that for the examination of the Central Midwives Board a somewhat higher standard of education will be required than has hitherto been necessary. She, therefore, urged that in face of the probability of candidates being refused a certificate on the ground of want of general elementary knowledge alone, without reference to their knowledge of nursing, county councils should select candidates who "can at least read and write with fluency and accuracy." The advice is sound; we are quite sure that in a very short time there will be no recognised midwives who cannot, at any rate, read and write fairly well.

### QUEEN ALEXANDRA'S MILITARY NURSING SERVICE.

We are officially informed that the following ladies have been appointed as staff nurses in Queen Alexandra's Imperial Military Nursing Service.—Misses A. B. Cameron, Wynberg, Cape Colony; H. M. E. Macartney, E. M. Rentzsch, S. O. Beamish, E. C. Armstrong, A. C. Mowat, E. M. Lang (to Lincoln for temporary duty), M. Antrobus, K. Roscoe, A. A. Steer, F. E. Davies, M. M. A. Copinger, M. Davis, C. D. E. F. Dunn, and H. Hartigan. Miss F. E. C. Watson and A. S. Wyatt have resigned their appointments as staff nurses. We are also informed that changes of station among matrons have taken place as follows:—Miss C. M. Chadwick, R.R.C., to Curragh on expiration of leave; Miss M. Russell, R.R.C., to Alton on expiration of leave; Miss M. Thomas, R.R.C., to Dover on return from South Africa: among sisters—Miss M. M. Blakely has been transferred to Egypt from Curragh; Miss A. Fitzgerald to Cambridge Hospital, Aldershot, on her return from Indian Troopship Service; Miss S. Lamming to Cadet Hospital, Royal Military Academy, Woolwich; Miss D. I. Rickards to Royal

Herbert Hospital, Woolwich, on expiration of leave; and Miss E. C. Humphreys to transport *Plassy* for Indian Troopship Service from Cambridge Hospital, Aldershot. The appointments of Miss H. M. Drage and Miss L. M. Toller as staff nurses have been confirmed.

### SCULCOATES GUARDIANS AND THEIR PROBATIONERS.

The Guardians of the Sculcoates Union have been trying to solve the problem why the nurses resign, and a proposition was made at their usual meeting for the appointment of a special committee to inquire into the causes and the administration of the Workhouse Infirmary. One of the guardians declared his conviction that the religious question is at the bottom of the difficulty. This view of the matter was, however, generally deprecated, and eventually it was decided to appoint a sub-committee of the house committee to go into the question and report. As there is some talk about differences between Roman Catholics and Protestants, and two Roman Catholics have lately resigned, it is desirable that the facts of the case should be known and dealt with.

### APPOINTMENT OF A NIGHT NURSE AT LOUGHBOROUGH.

The Loughborough Guardians have appointed a night nurse at the Poor Law Infirmary. This is well, but it would have been better if their action had been taken spontaneously, and not as the result of the report of the Local Government Board Inspector, who, on the occasion of a recent visit, found that there were 90 cases in the infirmary, attended to by two nurses, both on day duty. Many of these patients, the inspector stated, required much attention, 26 of them being bed-ridden. He also quoted figures showing that the number of deaths had steadily increased for three years, a fact which proves that more serious cases of sickness were being treated. The medical officer, he added, had urged the engagement of a night nurse, but the question had been allowed to remain in abeyance. However, the issue of this report has had the desired effect, and the 90 patients will, in future, have a night nurse to look after them.

### ST. PATRICK'S NURSES' HOME.

The interest which is felt in St. Patrick's Nurses' Home was shown at the annual meeting in Dublin by the large and influential attendance which included the Countess of Dudley, and the Archbishop of Dublin, who was in the chair. His Grace, in moving the adoption of the report, mentioned that the number of patients attended in 1904 by the fully-trained nurses and probationers was 2,142, the largest on record, while the number of visits paid exceeded 36,000. The staff, as he pointed out, is comparatively small and consists of a superintendent, six nurses, and five probationers, who are trained in connection with Queen Victoria's Jubilee Institute. The institution, which thus serves the twofold purpose of supplying trained nurses to attend on the sick poor of Dublin and its vicinity, and a training school for Queen's nurses, has now attained its majority; and the speeches delivered by the Archbishop and others conclusively demonstrate the increasing importance and the growing popularity of the work. Sir John W. Moore, the president of the Royal College of Surgeons, in paying a tribute to the work of the nurses, said that having lectured for some years to medical students and St. Patrick's nurses he thought that the latter were much more receptive than the average run of medical students. The reason for this was, he thought, that nurses when they began to attend the lectures came to them with a full knowledge of the responsibilities of their calling; but medical students had yet to learn these responsibilities.

### QUEEN'S NURSES AT WILLENHALL.

Generally speaking, the people in industrial centres respond more liberally to the appeals of nursing organisations for support than the people in market towns and cathedral cities. It is true that there may be more patients in industrial centres, but the sick poor in market towns and cathedral cities need as much attention as the sick poor in the heart of the Black Country. The fact that the Willenhall folks mark their appreciation of the labours of the Queen's nurses by contributing sufficient to cover the cost of the work, and to swell the amount of the balance in the hands of the treasurer, is very creditable to them. A charity football match yielded £28 7s. 11d., and a Saturday street collection £15 2s. 11d., but the number of annual subscribers, though not so large as required, is fairly satisfactory. The two nurses did not let the grass grow under their feet; they nursed nearly 200 cases, and paid upwards of 5,000 visits.

### CHESTER AND ITS NURSING ASSOCIATION.

At the annual meeting of the Chester and District Nursing Association, one of the members in seconding the adoption of the report, said that he was constantly hearing on all hands that the organisation "was absolutely unknown by the bulk of the people of Chester." Seeing that it was formed more than four years ago, that the staff consists of a superintendent and six nurses, and that last year the area of work was extended to a portion of Saltney by special arrangement, this is an extraordinary statement. If it be correct, the committee of the association are certainly largely to blame. Long before now they should have made it impossible for any householder in Chester to profess ignorance of its existence. At any rate, the financial support given to it is unworthy of an important city like Chester. The expenditure in 1904 was £743, as against £613 income, and the balance on the wrong side is therefore serious. But the unsatisfactory position is explained by the inadequate number of subscribers. Out of a population of 40,000, excluding Saltney, there are less than 200 subscribers. We agree with the Sheriff that there ought to be at least 500. The bazaar in Chester last week in aid of the funds was a substantial help, but the most essential point is to double, or treble, the number of subscribers, and secure a settled sufficient income.

### A NURSE'S WEDDING.

On Friday last Miss Maria Hayes, a nurse who was trained at St. Leonard's Infirmary, Shoreditch, was married at the Tabernacle in Burdett Road, London, to the Rev. Charles A. Schonberger. The bride was dressed in her indoor uniform, and the two bridesmaids, also nurses, wore the uniforms of their respective hospitals. The guests at the wedding breakfast included many nurses.

### The Nursing Outlook.

"From magnanimity, all fear above;
From nobler recompense, above applause,
Which owes to man's short outlook all its charm."

### NURSES BEWARE!

THE present position of nursing in England makes it bad for the public and also for the nurse. At the present time all the facts go to prove that the majority of the nurses who are supplied to the public have not obtained a three years' certificate from a recognised nurse-training school. Of course the larger proportion of such nurses have been engaged in nursing for a number of years, and they may or may not have acquired good knowledge of their duties and be in fact qualified to discharge them. These women have to live, and the knowledge of this fact has led many persons to exploit such nurses by establishing so-called homes or agencies which supply the public. The law courts have occasionally thrown some light on the profits made by the larger of these establishments which have proved to be very considerable. These profits, which ought of course to be shared with the nurses, are often retained by the proprietors, because the type of nurse we refer to has never had an opportunity to put herself right in a professional sense by passing an examination and obtaining a certificate which would qualify her to work under a nurses' co-operation or, in some public establishment where the nurse is justly and properly treated. The nurses referred to must obtain work in sufficient quantity to maintain themselves, and, being it may be without connection or influence, they readily fall into the hands of the private adventure agencies and homes, which often make considerable profits at their expense.

The evils and injustice of this state of affairs do not end with the fleecing of the nurses. The proprietors of such establishments in the ordinary course of business offer nurses to the public and extend their connection as much as possible. To meet the demand thus created they frequently take so-called nurses who may have had little or no training, a proceeding which is unjust to the better-qualified nurses, because it must cause the public and the medical practitioners to be often dissatisfied, and to regard all connected with such an agency as suspect, inefficient, or unworthy. Hence the necessity, as we pointed out a few weeks ago, for a system which would compel all nurse agencies and homes to be registered. A proper system of registration under the county councils, for instance, would insure that proper registers should be kept of the nurses employed, their training and qualifications, and of the cases, with the name of the nurse sent to each, and any material particulars relating to it. They would be under the inspection of the registration authorities and would have to conduct their business in such a way as to protect the public and the nurse from many of the abuses which at present attach to both.

Pending the enforcement of registration by legislation nurses who work for such homes and agencies must protect themselves by insisting upon entering into a business arrangement on commencing the work. Every nurse should secure herself by having an agreement with the proprietors, and in our view she would be well advised to never consent to a larger deduction than 10 per cent. of her fees, seeing that the experience of the Nurses' Co-operation shows that 7½ per cent. is the maximum sum which ought to be deducted, and that in practice 5 per cent. upon the earnings of a large staff provides a good margin of profit for the management. We are further of opinion, that so far as private nurses are concerned, it is better for them to stand out for their fees subject only to such commission, rather than to enter into an agreement to receive a fixed yearly salary with board and lodging between cases. The Co operation rule 7 provides that nurses must apply for fees and expenses before leaving the patient's house, using the account form supplied for this purpose. In view of a recent case in the law courts, it would seem to be essential that this practice should be adopted by all nurses, who should receive the fees and pay the commission due to the proprietor of the home on those fees as and when they receive them. No *boná-fide* proprietor of an agency or home could reasonably object to a practice of this kind, providing they entered into a proper agreement with their nursing staff, and were careful to have adequate references when each nurse was engaged.

Nurses who follow the plan here suggested will place themselves in a fair way to protect their own interests. With a view to help them to do this, the new edition of "How to Become a Nurse" will contain a form of agreement, and clear directions as to what each nurse should do, when entering into an engagement with the proprietor of a private adventure agency, or nursing home. In this way, we hope to render a real service to a multitude of hard-working nurses, who, in existing circumstances, often suffer injustice and wrong, which they might be spared by the adoption of business methods, when first commencing their duties as private nurses. No nurse ought to engage herself to an institution of the kind referred to without entering into a written agreement. This should set forth the terms and cover all points which it is important a nurse should cover to safeguard her own interests. Most of the present misunderstandings and losses could thus be avoided by nurses, and we hope they will take the lessons of the past to heart and be as business-like as possible in their own interests. In cases of doubt or difficulty we are always prepared to help nurses, as far as possible, on receiving a clear statement of the points at issue.

### Lectures upon the Nursing of Infectious Diseases.

By F. J. Woollacott, M.A., M.D., B.S.Oxon, D.P.H., Senior Assistant Medical Officer, Park Hospital, Metropolitan Asylums Board, Hither Green.

LECTURE XV.—SMALL-POX (*Continued*).

At the present time patients suffering from small-pox are nearly always removed to a hospital, if one is available, for the disease is so infectious that it can rarely be efficiently isolated in a private house. The sick-room or ward should be large and well ventilated, in order to dilute the infection and remove, as far as possible, the unpleasant and peculiar fœtor so characteristic of the disease. The patient should be protected from draughts and an even temperature of about 60° F. should be maintained.

In mild and especially in modified cases very little further treatment is required. Convalescence is established very rapidly, and shortly after the rash has appeared the patient may be able to leave his bed and take his ordinary diet.

At other times one of the chief difficulties in nursing arises from the character of the rash, and great care is necessary, particularly in confluent cases, in maintaining cleanliness. After the eruption has developed the patient cannot be bathed in the ordinary way, but the surface should be kept as clean as possible by frequent sponging. If there are many pocks on the scalp the hair should be cut short. As the pustules mature and rupture the clothing and bedding are constantly being soiled with pus, and should be changed as often as is necessary. As soon as the patient is strong enough, a warm bath should be given each day. Besides its cleansing effect it is very refreshing and tends to soothe the irritation. The presence of a large number of vesicles or pustules, together with the surrounding inflammation, of course renders the skin very tender, and it should be, as far as possible, protected from pressure and injury. The patient, especially when delirious, should be handled as gently as possible, and all the clothing and bedding should be soft and kept free from creases. The mattress should be soft and elastic, and preferably made of horsehair, and a water-bed may prove of service. Bed-sores are apt to form as the pustules rupture and must be prevented by frequent changes of position and protective dressings. In some cases the skin, especially on the hands and feet, is so exquisitely tender that pressure, however slight, causes acute pain, and a cradle should be employed to prevent the contact of the bed-clothes.

The food in the acute stage should be chiefly liquid and the patient should be encouraged to take as much as possible. Very frequently, owing to the presence of vesicles and ulceration in the throat, swallowing is painful and difficult, and in some few cases nutrient enemata may be necessary. As the temperature subsides and improvement begins, the appetite quickly returns, and the diet may be as quickly increased. Throughout the illness it should be nourishing and plentiful, and alcoholic stimulants are often beneficial.

The attempt may be made to keep the temperature in check by sponging, wet packing, or the ice-cradle; but owing to the state of the skin, these methods of treatment are not always effectual.

In the acute stage, while the rash is appearing and for some time after, there is usually more or less delirium. It is always present in confluent cases, and is not infrequent in comparatively mild cases. The type of delirium is very characteristic. The patient gets but little sleep, is restless and somewhat excitable. He is constantly attempting to get out of bed, often asks for his clothes, says he feels quite well, and is obviously ready to seize the first opportunity of escaping. At times he may be plausible and cunning in order to throw the nurse off her guard. Under these circumstances he must be watched with the most unremitting vigilance. The nurse should never lose sight of him, even for a moment, and if necessary the door must be locked and the windows barred. The attempt should be made to quiet him by conversation, and he should be humoured in every possible way. Dr. MacCombie states that "a useful device is to dress the patient in a dressing-gown stockings and slippers, and allow him to walk up and down, the nurse holding his arm, and at the same time keeping her eye on the door and window. Very often the patient, after a few turns, finding that he is unable to continue the exercise, is glad to get back to bed." Occasionally the delirium assumes a violent form, and the help of a male attendant may be necessary. To some extent the struggles of the patient may be restrained by a sheet passing round the chest and fastened to the sides of the bed, but for the most part mechanical restraint is unsuitable, as it will almost certainly result in considerable injury to the inflamed skin; and only when it is absolutely necessary for the safety of the patient should force be used.

During the stage of secondary fever the delirium is of a different type and similar to that seen in enteric fever. As a rule the patient is too weak and exhausted for any attempt at escape.

There is no method yet discovered by which the full development of the small-pox eruption can be prevented and, unless they are modified by previous vaccination, the pocks will go on to maturity in the way described in the last lecture. It has been claimed that, if patients are treated from the first in a room to which only red light is admitted, the rash will be to some extent checked; and the attempt has been made to prevent the formation of pus by early and careful cleansing of the skin and the application of antiseptic dressings. Both these methods are difficult to carry out efficiently, and even when efficiently carried out, usually prove failures. Our treatment of the rash is therefore reduced to relieving as far as possible the pain and discomfort, and dealing with any urgent symptoms as they arise.

Up to the beginning of the pustular stage there is very little that can be done, but during that stage the pocks are practically converted into a number of small superficial abscesses and may be treated as such. The tops of as many as possible may be snipped with curved scissors, the exuding pus wiped away and boracic fomentations applied. Unfortunately the method of treatment is tedious and only a small part of the body can be dealt with at one time; but it certainly relieves the pain and tenderness and is well worth trying. In other parts, where the pustules rupture spontaneously, the separation of crusts may be assisted and the inflammatory swelling reduced by the use of poultices or fomentations. If the hands and feet are swollen and painful, the thick, hard skin should be softened by soaking in warm water and then fomented, and the escape of pus may be assisted by the use of the scissors or knife.

The irritation of the skin over the whole surface of the body is often very troublesome, sometimes amounting to torture, so that the patient cannot rest or sleep, and, unless restrained, will scratch and tear at his skin in order to obtain some respite from suffering. In severe cases the irritation can never be entirely relieved, and a certain amount of scratching is inevitable. It should, however, be prevented, as far as possible, by cutting the finger-nails quite short and, if necessary, muffling the hands, for it is sure to result in deep and unsightly scarring. More or less relief may be given by the use of various oils, ointments, powders or lotions, but their action is unreliable and they often prove all but useless. Cold compresses, frequently changed, occasionally prove beneficial. Special attention should be paid to the face, and whatever treatment is ordered should be applied on a mask of lint, with holes cut in it for the eyes and mouth, and carefully bandaged in position.

As might have been expected the presence of many pustules often sets up various kinds of inflammation and suppuration in the adjacent parts. They usually appear in the scabbing stage and take the form of boils or subcutaneous abscesses, which may at times be very numerous. In some cases cellulitis results so that the skin itself and the tissues beneath become acutely swollen and inflamed, and deep abscesses may appear at some distance from the surface. Whatever form is assumed the treatment is much the same. The abscess is opened by the knife and if necessary drained, and boracic fomentations are applied.

A more serious complication is erysipelas, which occasionally attacks the skin. The patient must be promptly isolated, for, unless this is done, others in adjacent beds are almost certain to be infected. The clothing and bedding of the patient must also be thoroughly disinfected.

The state of the mouth and throat is often a source of much discomfort. They should be kept as clean as possible by means of antiseptic and soothing washes and gargles, and if necessary by syringing. The nose needs similar treatment; and when scabs form round the lips and the nostrils they should be softened with oil and removed as soon as possible. Sucking ice is occasionally useful, especially if the tongue be much swollen. If the inflammation passes along the Eustachian tube it may implicate the middle ear, and cause pain and subsequently ear discharge.

More or less hoarseness of the voice is common and needs no special treatment. If the cough become croupy and the breathing obstructed, the use of the steam-kettle affords relief, but at times tracheotomy may be necessary. Unfortunately recovery rarely follows the operation, for the patient is usually suffering from a very severe confluent attack under which he succumbs.

The eyes need very careful attention throughout the disease, as the eyelids are often much swollen and discharge collects beneath them; while the conjunctiva is inflamed and swollen, and occasionally a pock may appear on its surface. They must be kept as clean as possible by frequent bathing with warm boracic lotion, and vaseline should be applied to the edge of the lids so that they do not become glued together. The inflammation and pain may be relieved by fomentations. Not infrequently, especially in the more severe attacks, ulceration of the cornea occurs. Usually the ulcer is small and may be cured by the use of atropine drops to dilate the pupil and the application of the yellow oxide of mercury ointment; but occasionally it is large and deep and may lead to sloughing. In these severe cases the interior of the eye may become involved, so that the sight is destroyed, while the eyeball itself is converted into a bag of pus and must be removed by operation.

### Nursing in a Typhus Epidemic.

BY AN OCCASIONAL CORRESPONDENT.

During the typhus epidemic in Lancashire a few years ago, there were four of us specially set aside for night duty. Of course we had volunteered to go, as our matron never sent any of us to any disease that did not come in the ordinary course of infectious training without we consented and were fully prepared to take the risk. The lower wards, sometimes used for small-pox, happened to be empty, so were utilised for the typhus cases. We were strictly isolated, an empty ward being fitted up temporarily as a nurses' home. The cases came in very fast for a fortnight, the ambulance arriving as late as midnight with two or three patients at once, not always from the same house, but from the courts in different places where cases had been notified. Of course the outbreak was amongst the lowest of the lower classes; people who did not live but merely existed in dank cellars, which more often than not contained more than one family.

### Dirt and Typhus.

To show how easily these people go back to their dirt after a period of cleanliness and regularity in hospital, I may state that a lad was admitted one night in an absolutely filthy condition, to the ward in which I was on duty. How he came to be sent in in the first instance was a mystery to us, as there was nothing the matter with him except an appalling condition of filth. A nice bright lad of 18 he was, too, when we had got him clean, and he helped us in many ways in the ward during the mornings. For although he had not got typhus, we were obliged to keep him a certain number of days in case he developed it. So he was put to bed for a time and not allowed to go near the other patients. As the infection only carries 7 feet there was no fear of him catching the fever unless he had it already in his system. However, in less than a month he was discharged and went home. Matron gave him a suit of clothes, those in which he had come in having been necessarily burnt. Before he went I shook hands with him. "Good bye, Walter," I said; "I hope you will keep all right, and not come back to us shortly. Try and keep yourself clean and tidy now. You look quite smart." He blushed, and we could actually see the blush—which would have been an impossibility a few weeks previous—and he departed. In three weeks' time he came back again. He had gone home to the same dirty dark cellar, and had really got the fever this time. As I helped to carry him in, the poor delirious head rolled about, and the face was quite as deplorably disfigured with dirt and vermin as it had been before. The clothes which matron had given him were certainly not worn by him. He left us cured and clean once more, but the case illustrates the difficulties of impressing upon a certain class of people the desiraibilty of cleanliness.

### Nearly 200 Cases.

During one month we had nearly 200 cases, of which only twelve died. Their temperatures were 106° and 107° F. on entering, and eight out of the twelve might have recovered if they had not been heavy drinkers. But with the height of fever delirium tremens set in and we knew that they could not fight both. It was no unusual thing during the first few days to be met when going on duty with the words, "So-and-so is dying fast, I don't think she can last till midnight;" "Such-a-one may possibly live till morning, but it is doubtful;" and, "Mrs. —— has Cheyne-Stokes' breathing, so give her as much attention as you can," which meant that three of the twenty were in such a condition that they might go at any time. The wards were

INCIDENTS OF TYPHUS NURSING—*Continued.*

constructed to hold 32 beds, 16 in each division, but they had been cleared of a number, for they now contained 20, having 10 in each part, divided by a kitchen. A regular gale blew through the wards, and every window was open top and bottom as far as it would go, the draught almost raising the bed-clothes from the patients. This was a great comfort to them, although a good many of them had ice-caps, but it was by no means pleasant for the nurses. We were scarcely able to keep the fires in the stoves from being blown out; however, fresh air was the only safeguard against contagion which we could employ. Moreover, we were very short-handed, some of the nurses having gone for their holidays. We had several temporary nurses in, but matron kept them in the top scarlet-fever wards, preferring that her own nurses should have the typhus experience. Twenty of these cases were as many as we could manage, although we were used to 32.

### The Class of Patients.

At one time 18 out of my 20 had seen the inside of a prison more than once. This shows the class of person we had to deal with, but when they were convalescent I never had such grateful patients in the whole course of my nursing experience. We often found the very poorest and lowest the most grateful. They were quickly convalescent, for after the crisis, which occurs on the 16th day, unless there are complications, the patient's temperature drops from 104° or 105° to 98°, heavy perspiration sets in, and they sleep so soundly that there is the greatest difficulty in arousing them even for nourishment. This continues for a few days, and then the patients are practically convalescent; the only thing to be feared being rheumatism in the joints. Any joints with this tendency were wrapped in cotton wool immediately. Most of our patients were fit to go home in six weeks.

### A Dangerous Experiment.

For the first three weeks of the epidemic, matron was seriously concerned about two of us who were in the acute wards on night duty. I expect she made as much fuss about the day nurses, but, of course, we did not know. Every night when she came on her round, she inquired most anxiously about us, and we were told to be sure and report to her any night before going on duty if we did not feel well. However, all went well with us for a fortnight, and then one evening, just before going on duty, the nurse in the ward adjacent to mine, told me that she felt fearfully ill. My own head had been going like a sledge hammer, so that I had scarcely slept all day. Patients had informed me that just before they lost their reason, the sounds in their head resembled that of clocks striking; so we were afraid that we might be down with fever ourselves as matron had dreaded. We made up our minds to dose ourselves with quinine, which we took in enormous quantities. I know a doctor would never have ordered a patient a quarter as much, but I believe it checked the disease. As it was, I think we had it suppressed. Neither of our temperatures were higher than 103°, and that not for long, which may have been due to the amount of quinine we had taken. When we began to feel slightly deaf we lessened the quantity, but for ten nights we were both awfully scared lest matron or doctor should find us out. But as we did not complain, and could get through our work, they did not pay attention to us; and certainly they had enough to do to look after the patients. Of course, if we had fallen victims it would probably have gone very hard with us, because we did not give in at the first. My colleague died six months later from typhoid fever, caught from a patient, a baby, in her ward. Her death cast quite a gloom over us all for some time.

### The Anglo-American Nursing Home at Rome.

The annual meeting of the Anglo-American Nursing Home was held on Wednesday last week at the British Consulate, Mr. A. C. Trench being elected chairman. The minutes of the general meeting of January 1904 were read and confirmed, and referring to the report of the managing committee for the year 1904 the committee states that it "feels that the favourable result of the year's working is a matter for sincere congratulation, especially as in pursuance of its policy of carrying out the intentions of the supporters of the home with regard to an adequate supply of trained nurses, it increased the number of its staff from 12 to 16, and thereby ran some risk of having to face an increased deficit at the end of the year." The patients treated in the home, from January 1st to December 31st, 1904, was 35—22 medical and 13 surgical. Eighty-five private cases were nursed. At the height of the season the staff of nurses proved insufficient, and applications for their services could not be complied with. The final payment in connection with the purchase of the home had been made, so that the property was now finally vested in the name of the association. The plot of land for the extension of the home had been purchased, and special appeals are being made by the committee to raise the necessary funds. In conclusion the committee warmly thanks "the physicians and surgeons who have most kindly given their professional services to free-bed cases"; and also "expresses its sincere appreciation of the directress zealous activity in the management of the home, and of her constant efforts to increase its utility."

Miss Bulwer inquired how many non Anglo-Saxon paying patients were treated in the home, and the chairman replied that the number was two, but failed to understand the object of the inquiry, he said, till it was explained to him that thus alone would the public be able to test how far the institution was patronised by those for whose benefit it was founded. She also asked how it was that 16 nurses in 1905 had each earned on an average 700 lire less per head than seven nurses in 1901. The chairman replied that work fluctuated, and only broad results could be considered. Miss Bulwer then alluded to the statement that an Italian woman, Signora Jacobini, whose doings filled the papers under the heading "*Lo Scandolo Bucci*," had been an inmate of the home from November 1903 to April 1904, which was not contradicted.

Miss E. C. Vansittart inquired why the debts of the home had grown from 3,149 15 lire to 5,682.45 lire? and the chairman replied that this caused no anxiety as there was always money outstanding in every concern.

When the election of new members was being proceeded with Miss Bousfield and Miss D. E. Bulwer asked to withdraw their names from nomination as they "found it impossible to serve on a committee which refused to investigate cases freely." After four new members had been elected, formal votes closed the meeting.

### Death in Our Ranks.

The whole staff of Stoke-upon-Trent Union Hospital mourn the loss of probationer Nurse Mabel Ecclestone, who died after six months' illness, on Friday last. She was only 24 years of age and her gentle and loyal disposition had gained for her the esteem and affection of the patients and the staff.

### A Stage-Army "At Home."

A MEETING of protest against the license of the Board of Trade being granted to a society which sought to obtain absolute authority over trained nurses was held at 20 Hanover Square on Wednesday afternoon, under the auspices of the Matrons' Committee of the Society for State Registration of Trained Nurses. There were present on the platform Lady Helen Munro Ferguson in the chair, Miss Isla Stewart, Mrs. Bedford Fenwick, Miss Mollett, Miss E. S. Haldane, Miss Mary Burr, Miss G. A. Rogers, Miss Christina Forrest, Miss E. M. Roberts, Miss Ross, Miss E. M. Musson, and other members of the Society for the State Registration of Trained Nurses.

It was quite one of the pleasantest gatherings of nurses we ever attended. Everyone seemed to know everyone else, and the noddings and bowings seemed to show plainly that friends of long standing had met in a kind of family party to discuss a family matter. As the meeting progressed this family character was confirmed by the fact that when each resolution was put the chairman did not trouble to invite any of those present to vote against it. Indeed, the attitude assumed by the speakers was one of certainty that the meeting was unanimous and needed no convincing.

Lady Helen Munro Ferguson said that the object of the meeting was to give expression to a protest already entered by the Matrons' Council of the Society for State Registration against a society which, while ostensibly promoting the objects for which the family had worked so long, would in reality fetter for all time its further action and development. The status and internal government of the profession was threatened, and matrons and nurses must make their voices heard. Although there might perhaps, among the unknown twelve who sought to form an autocracy in the nursing world, be found room for one or two nurses and doctors, there was nothing in the articles of constitution to ensure this; indeed, so completely had the element of representation been banished that it read more like a conciliatory proposal from the heads of a country governed by an autocracy. It was an instance of the rapidity with which public opinion was turned in favour of State registration, but it was not enough to turn, the turning must be made in the right direction. It was as if the Gods had come down from Olympus, and devised a scheme so simple that everybody could understand it, and which only needed incorporation by the Board of Trade to be a success. While the authors of the scheme kept most modestly in the background, it might be thought, from the names which did appear, that it emanated from the Bank of England. Yet she could not suppose that the nursing profession had become so important to the Bank of England as this.

Next came Miss Isla Stewart. Her main objections were:—(1) The registration ought to be by Act of Parliament; (2) its unrepresentative constitution; (3) the question being under consideration in the House of Commons, the time was inopportune to introduce it. She proposed the following resolution:—

> That this meeting of hospital matrons and trained nurses emphatically protests against the attempt now being made by seven gentlemen in the City of London to obtain the license of the Board of Trade to incorporate a society which seeks for authority to organise the professional education of trained nurses, to exercise disciplinary powers, and to control their work.
>
> This meeting is strongly of opinion that no such powers can be usefully or successfully exercised except by a body composed of professional persons upon which trained nurses have direct and sufficient representation.

Miss Mollett seconded, from the nurses' point of view, owning to irritation, and asserting that, while nurses were thoroughly dissatisfied with their status and want of curriculum, the public was sick of hearing these matters discussed. She stated that nurses must arrange their affairs for themselves. In England, if you were only helpless enough, and made a big enough muddle, you might be perfectly certain that someone would come along and make you a little more helpless, wash your face for you, brush your hair, and tie your pinafore behind. It was "monstrous and calumnious." Personally she liked to manage things herself, and the family clapped to show that they did too.

Mrs. Bedford Fenwick supported the resolution, and was also historical. The secreted charter of the R.B.N.A. claimed some attention, and there was a good deal about philanthropists, who were all very well as long as they kept to their own sphere. There were people who had subjected themselves to a great deal of mental and physical strain during the last 17 years, in order to promote State registration, and were they going to sit down under a proposal emanating from the opposition camp? No! There was no desire to reflect upon the motives of those financiers who had brought forward this scheme for "our benefit and happiness;" but as it emanated from Threadneedle Street, it would be wise to look at the financial aspect. Were they going to pay? Oh, no. The woman paid, the nurse paid, and through the nose. But a worse thing still was judgment and condemnation without power of protection, a most un-British thing; and there was absolutely no clause in the whole of the memorandum giving the nurse power to protect herself against the decision of the promoters of this proposed register. Finally, there was a foreign element in the signatures. Did Mrs. Bedford Fenwick mean to suggest that the scheme was "made in Germany"? However this might be, the family wasn't going to stand it.

Miss Haldane and Miss Mary Bull supported, facing this new peril with great fortitude, and calling upon the family to stand by Mr. Ferguson's Bill. That resolution passed, another was proposed:—

> That this meeting earnestly supports the Bill for the Registration of Trained Nurses, introduced into the House of Commons by Mr. Munro Ferguson, being convinced that the only possible remedy for the present disorganised condition of nursing education in this country, and the many evils now existent in the nursing world, would be the institution of a representative Nursing Council, empowered by Act of Parliament to supervise nursing education, and maintain common rules of discipline in the nursing profession.

Miss Rogers, in moving, was not sure that the new society would not really play into the hands of U.S. At any rate, the appointment of an examination board showed how much it was needed to be done. It appeared to her that financiers, being large subscribers to hospitals, in managing this society, would have the power to shut the door on those nurses who did not hold their certificate. Miss Forrest feared it meant the "unaproning of the nurses by gilded dummies," and Miss Musson talked of "Hokey-pokey" management.

Yet another resolution:—

> That copies of these resolutions be sent to the Chairman of the Select Committee on Nursing, and that a deputation be now appointed to wait upon the Right Hon. the President of the Board of Trade (if he will receive the same), to present to him copies of these resolutions.

And then Lady Helen Munro Ferguson, Mrs. Bedford Fenwick, Miss Isla Stewart, Miss Haldane, Miss Huxley, and Miss Peter were proposed as a deputation to the Board of Trade, and the family party broke up and said Good bye.

### Central Midwives Board.

### THE QUESTION OF POOR-LAW INFIRMARIES.

An adjourned meeting of the Central Midwives Board was held at the Board-room on Thursday, February 16th, to continue the consideration of the general principles upon which Poor-law institutions should be recognised. Dr. Champneys was in the chair, and the other members present were Dr. Cullingworth, Miss R. Paget, Miss Wilson, and Mr. Parker Young.

The Secretary informed the Board that he had applied to the Local Government Board for the orders relating to midwives on behalf of Miss Wilson (one of the Privy Council nominees), but had received a reply to the effect that it was not their practice to comply with such requests.

Dr. Cullingworth moved and Miss Wilson seconded the following resolution, which was carried:—

"That no Poor-law Institution be approved as a training school for midwives unless the average number of deliveries per annum be sufficient to insure the training of at least three midwives."

It was moved by Miss Paget, seconded by Mr. Parker Young and carried:—

"That no application on behalf of a Poor-law Institution to be approved as a training school for midwives be accepted unless it is in the form of an official application, signed by the Clerk to the Board of Guardians."

Suggestions as to the lines upon which the Board's Inspector should make inquiries were discussed, and it was agreed that a draft copy of these should be sent to each member of the Board for consideration.

### Appointments.

[No charge is made for announcements under this head, and we are always glad to receive, and publish, appointments. The information, to insure accuracy, should be sent from the nurses themselves, and we cannot undertake to correct official announcements which may happen to be inaccurate. It is essential that in all cases the school of training should be given.]

Chester Union Infirmary.—Miss L. R. Jones has been appointed superintendent nurse. She was trained at Mill Road Union Infirmary, Liverpool, and has since been Sister at Walton Workhouse Infirmary, West Derby, Liverpool.

Dumfries and Galloway Royal Infirmary.—Miss Lilian Arrowsmith has been appointed night sister. She was trained at Ancoats Hospital, Manchester, and has since been charge nurse in the same institution.

Liverpool Lying-in Hospital.—Miss Jessie Brown has been appointed Matron. She was trained at Edinburgh Royal Infirmary and Clapham Maternity Hospital. She has since been district nurse at Dalbeattie, has done private nursing, has been assistant matron at one of the Concentration Camps in South Africa, matron of the Battersea district branch of Clapham Maternity Hospital, and district, private and visiting nurse at Tangier, Morocco. She holds the L.O.S. certificate.

Southwark Infirmary, East Dulwich.—Miss Millicent Violet Ryott has been appointed night superintendent. She was trained at the Bradford Royal Infirmary, and has since been attached to the Bradford Nurses' Institution. She has also been sister at the Royal County Hospital, Ryde, Isle of Wight, and sister at the Southwark Infirmary.

### Novelties for Nurses.

By Our Shopping Correspondent.

### A NURSE'S CLOAK.

The Hospital Contractors and Nurses' Outfitting Association, Stockport, have sent a nurse's cloak for inspection. It is of navy blue cloth, tailor made, with a detachable cape, and the price is 22s. 6d. It is one of the many cloaks supplied by the association, and the firm draws particular attention to the fact that all cloaks are made by them—no factory goods under any circumstances whatever being stocked — and each cloak is made for each individual customer. Matrons' and nurses' woollen and cotton dresses are also made to measure, the former from 24s. 6d., the latter from 13s. 6d. complete. The firm also makes a speciality of nurses' bonnets, which can be had trimmed from 5s. 11½d. Nurses should write to the association for their new illustrated catalogue which gives full information of the many requisites supplied.

### THE ATHEENIC UNDERWEAR.

Atheenic is the trade name for all the various grades of elastic silk, silk and wool, and pure wool materials which are manufactured and made into undergarments at the Atheenic Mills at Hawick in Scotland. Purchasers are invited to apply direct to the mills and procure the garments at the prices which are published in the price list. These prices would be extremely moderate for quite ordinary materials, and as the Atheenic materials are of exceptional excellence they are more especially so. The sample patterns issued from the mills are most fascinating and tempting. The finest and softest of silk and wool stockinette with many intermediate grades of thickness up to the heaviest and closest material make it possible to select for all weathers and climates. The soft and inviting texture with its even and smooth surface exhibits the perfection of manufacture which is further carried out in the carefully shaped and finishing of the garments. The seams, strong and unobtrusive, are sewn together by machinery, and reveal none of the inequalities which must occasionally occur in connection with hand-sewn garments. Our readers would certainly do well to write to the mills for a price list.

### TRAVEL NOTES AND QUERIES.

By our Travel Correspondent.

Paris for 4 or 5 Francs a Day (Matron).—I do not quite understand your question. Do you mean that the above sum should cover food as well as lodging? If so, I must tell you that such terms are impossible in Paris, if the surroundings are respectable. You say the ladies would not like a second-rate hotel, but not even a third-class house would accommodate them on such low terms. Paris (for a short stay) is expensive, and there are many "extras." The cheapest hotel I know of has rooms on the fifth floor for 1 franc 50 per night, but food, light, and attendance would bring costs up to 8 francs a day. Write me again more fully. The ladies do not want to start till May and I will in the meantime make inquiries as to cheap accommodation, but believe me 5 francs per day is too low for any Paris hotel. Fare from Boulogne to Paris about 14s. single second class, and return 25s.

Rules in Regard to Correspondence for this Section.—All questioners must use a pseudonym for publication, but the communication must also bear the writer's own name and address as well, which will be regarded as confidential. All such communications to be addressed "Travel Correspondent, 'Nursing Section of *The Hospital*,' 28 Southampton Street, Strand." No charge will be made for inserting and answering questions in the inquiry column, and all will be answered in rotation as space permits. If an answer by letter is required, a stamped and addressed envelope must be enclosed, together with 2s. 6d., which fee will be devoted to the objects of "The Hospital" Convalescent Fund. Ten days must be allowed before an answer can be published.

### Everybody's Opinion.

[Correspondence on all subjects is invited, but we cannot in any way be responsible for the opinions expressed by our correspondents. No communication can be entertained if the name and address of the correspondent are not given as a guarantee of good faith, but not necessarily for publication. All correspondents should write on one side of the paper only.]

### ULTIMATUM OF ASYLUM NURSES.

"LITTLE MARY" writes from Borough Asylum, Ryhope, Sunderland: Will you kindly allow me to correct the statement which appeared in last week's HOSPITAL regarding the coming on duty of the Sunderland Borough Asylum nurses. It must be admitted that some (not all) have been rather negligent in that respect; but it is quite untrue that the patients have ever been left without a nurse or attendant, as the case may be. The night nurses do not go off duty until 6.45 A.M., and patients under special observation are under no circumstances left night or day.

[If our correspondent's statement be correct, it is a pity that the facts of the case were not accurately set forth at the meeting of the Sunderland Corporation, when the matter was discussed.—ED. THE HOSPITAL.]

### AN OVERWORKED NURSE AT EPSOM.

THE President of the Epsom District Nursing Association writes:—Referring to the note in your issue of February 4th, headed "An Overworked Nurse at Epsom," I beg to state that the district nurse alluded to as "one nurse to 11,000 inhabitants," attends to very poor, only those who are unable to pay a fee—Epsom being a prosperous town, these are limited in number. The nurse's visits average 10 per day, which is not excessive; she has one month's holiday per year, when a locum tenens has always taken any of the cases requiring attention. Nurse Axon has worked for 15 years in Epsom, and is greatly beloved by the poor; her salary, board, and washing amount to £105 per annum. There are a number of nurses working in Epsom who attend those able to pay a fee, therefore the district nurse does not interfere in any way with those who are gaining their livelihood by nursing.

### WOMEN INSPECTORS OF MIDWIVES.

"W." writes: I think everyone will agree with "L. M. B." that a district nurse who undertakes general nursing should be fully trained, but where only midwifery is required I cannot see where the necessity for general training comes in. Most midwifery training schools very strongly object to their graduates undertaking general nursing, and for my own part I have always regarded it as quite a breach of professional etiquette to do so, or even to give an opinion on a case not strictly in my own department. I am afraid "L. M. B" has been singularly unfortunate in the class of trained midwife she has met. It must be a very lax training school which will give a midwifery certificate to a nurse "with only a meagre knowledge of the rudiments of maternity nursing," and a committee which will appoint an ignorant woman of that sort is very much to blame, and must be responsible for much needless suffering and many deaths. In the Rotunda Hospital where I trained we were obliged to pass a stiff examination before getting a certificate, and to show a thorough knowledge of our work, and of every possible complication of pregnancy, labour, and the puerperium, as well as the care of babies and all infantile disorders. At the same time, although the midwife is expected to detect any abnormal symptoms, neither she nor even a fully-trained nurse is to treat such. A nurse's duty is to report at once to a doctor, and let him make the diagnosis and prescribe accordingly. I give my own routine as presumably that of any trained midwife. If during my morning visit to a patient I find any abnormality about the temperature, pulse, lochia, or soon, I take a note of every detail of the case, and report immediately to the medical officer under whom I work. I may know perfectly well what is the matter and what he will order, but it is not my business to diagnose. He does that, and if necessary visits the case himself. Of course if the doctor is not available for several hours, I have been taught what to do in the meantime, or if it is very urgent I apply to another doctor, and then mention what I have done to the medical officer as soon as possible. I should think that is the usual course pursued by all nurses, maternity or general. With regard to inspectors of midwives, I think that "L. M. B." does not quite see my point. I said that where education and other things are equal, a woman who has directed all her energies for a given number of years to midwifery alone is better qualified to inspect midwives than one who has divided the same time among several branches of nursing, giving perhaps a few months or even a year to midwifery. Midwifery is a speciality, and a specialist in any line is considered superior to a general practitioner. I do not mean to say that every midwife is qualified to be an inspector, but neither is every fully-trained nurse qualified for the higher ranks of her branch of nursing. The upper grades of any branch require a great many qualities, not necessarily acquired in any kind of training. It seems to be rather a slur cast on the certificates of the London Obstetrical Society and Central Midwives Board, and of our large training schools if those holding them are not fit for the higher grades of their profession without any further training. Another thing I must remark is that as "L. M. B." mentions the cost of general training, midwifery is not to be had for nothing. The Rotunda Hospital fee is 25 guineas for six months, and it is not the most expensive, nor do the probationers receive salaries during training as they do in some general hospitals.

### A New Book for Nurses.

AN ELEMENTARY TREATISE ON THE LIGHT TREATMENT FOR NURSES. By J. H. SEQUEIRA, M.D. (London: Scientific Press, Limited. 1905. 83 pages, with eight illustrations. Price 2s. 6d. net.)

DR. SEQUEIRA, physician in charge of the skin department and lecturer on dermatology at the London Hospital, has written an excellent text-book for nurses on the light treatment. In the preface he states that care in the details of the treatment is essential to success; and that to make these details intelligible to the attendant it is important that the principles underlying them should be thoroughly understood. In straightforward, concise language he has explained these principles. The lines followed are those of Finsen's Light Institute, in Copenhagen, with the modifications suggested by Dr. Sequeira's experience in the London Hospital. The first chapter on "Light," gives a description of the sun's rays, visible and invisible; also the rays given by gas and candle-flames, electric arc-lamps, and incandescent electric lamps. A chapter on the influence of light upon living organisms includes a pithy paragraph on the influence of light upon Bacteria. There is a good description of the apparatus for treatment by the chemical rays of light; and the technique of the Finsen treatment is dealt with at length. Great stress is laid on the importance of the disinfection and cleansing of the compressors, and the precautions to be taken by the nurse. The book, which is well printed in large type, is illustrated by photographs taken in the Light Department of the London Hospital, and various diagrams. It should be of very great service to nurses.

### To Nurses.

### Movements of Royalty.

ON Saturday the King and Queen, accompanied by Princess Charles of Denmark, drove from Buckingham Palace to Cadogan Square to visit Princess Louis of Battenberg, previous to her departure from London to Russia. The Princess Louis is an elder sister of the Grand Duchess Sergius, whose husband was murdered in the streets of Moscow on Friday afternoon. On Saturday evening the Princess left London in order to attend the funeral of her brother-in-law. The King on Sunday afternoon visited the Russian Ambassador and the Prince and Princess of Wales also called at the Embassy.—On Monday it was officially announced that the King had commanded that the Court shall wear mourning for one week for the Grand Duke Sergius; but it was intimated that ladies who had already provided themselves with dresses for the Court this week need not appear in Court mourning.

### The Royal Gift to Ireland.

THE Prince and Princess of Wales have given great pleasure in Ireland by presenting to the Irish people, in remembrance of their recent visit to the sister island, five of the chief works in the Forbes collection, now being exhibited at the Royal Hibernian Gallery in Dublin. Three of the pictures are by Constable and two by Corot, and it is hoped that the kind action of their Royal Highnesses may have the effect of inducing some of the wealthier inhabitants of the city to follow their example, and thus to secure the desired establishment of a gallery of modern art in Ireland.

### Betrothal of the Duke of Saxe-Coburg.

IT is announced that Duke Charles Edward of Saxe-Coburg-Gotha, son of the Duchess of Albany, is betrothed to Princess Victoria Adelaide of Schleswig-Holstein Sonderburg-Glücksburg, niece of the German Emperor. The Princess was born at Grünholz on December 31st, 1885, and is 18 months younger than the Duke. The Duke is at present serving as a lieutenant in the 1st Regiment of Foot Guards at Potsdam, where he occupies, with his mother, a handsome villa on the outskirts of the town. No date has yet been fixed for the marriage, but it is understood that after the ceremony the young couple will take up their residence at Coburg. The German Emperor telegraphed the announcement of the engagement to the King and Queen.

### Murder of the Grand Duke Sergius.

ON Friday the Grand Duke Sergius was murdered in the heart of Moscow. He was driving towards the Kremlin Palace when a bomb was thrown under his carriage. The latter was blown to pieces and the Grand Duke was instantly killed, his head and his limbs being torn from the body. The driver also received fatal injuries. The assassin, who is in custody, is said to be a man of education. The Grand Duke Sergius was the uncle of the Tsar, brother-in-law of the Tsaritsa by his marriage in 1884 with Princess Elizabeth of Hesse. He was born in May 1857, and from 1891 until a few weeks ago was Governor-General of Moscow. On Saturday an Imperial manifesto was issued by the Tsar in which his Majesty declared that the whole life, activity, and care of his uncle and friend were constantly devoted to the service of the Fatherland.

### Appeal of Russian Women to the Tsaritsa.

AN address has been presented to the Tsaritsa, signed by a large number of Russian women, the mothers, wives, and sisters of the men who are fighting in the war in the Far East, in which they express their ardent desire for peace. "Mothers' hearts," they say are breaking, "and they cannot remain silent." They "feel that many more lives will yet be sacrificed," and they ask the Tsaritsa to be their pleader, and to pray her husband that he may listen "to the voice of the country and the cry of its mothers." In conclusion the appeal says, "If the Tsar leads the country into the paths of greatness, its women will help in the work of its organisation by guiding their brothers and children into the new way and a life of light."

### Prospects of Peace in the Far East.

THERE is no news of importance from the seat of war in the Far East, but a telegram from St. Petersburg states that Russia is prepared to consider terms of peace which shall include the recognition of Japanese ascendency in Korea, the cession to Japan of Port Arthur, and the Liau-tung Peninsula, and the restoration to China of a part of Manchuria. It is added that the only difficulty is as to the amount of the indemnity on which it is understood that Japan will also insist; but this is not regarded as insuperable.

### Lamentable Accident on a Submarine.

A DISASTROUS accident happened on Thursday last week to a submarine at Queenstown. The vessel was lying in the harbour, where it was about to be employed in demonstrations before a number of military officers, when a violent explosion occurred on board. Most of the officers and crew were below at the time. A rescue party was at once despatched from a gunboat lying alongside, and while they were at work a second explosion took place. A sub-lieutenant and three men were killed at once, and another officer and several men were more or less injured. The King, on hearing of the event from the Secretary of the Admiralty, at once sent a message of regret and sympathy. It will be remembered that an explosion occurred on a submarine at Portsmouth some time ago.

### A Lady Water-Colourist.

MISS EMILY MURRAY PATERSON, whose private view of Dutch and Venetian Waterways took place on Saturday at McLean's Gallery, is in the first rank of water-colourists in Scotland. Some time ago she was elected a member of the Royal Scottish Society of Painters in Water-Colours—an honour not often bestowed upon ladies. Miss Paterson is a native of Edinburgh, where she has exhibited much of her work. She has travelled widely, and has found many of her subjects in Holland, Italy, Belgium, Norway, and Switzerland. In Switzerland some of her sketches have been made at an altitude of 10,000 feet. A sketch of the *Hohenzollern* in the Grand Canal, Venice, taken at the time of the German Emperor's visit last year, is included in the present exhibition. A previous picture by Miss Paterson of the Imperial yacht painted in Norwegian waters is now in the possession of the Emperor.

### New Play at St. James's Theatre.

WHILST Mr. Alexander is resting the latest production at the St. James's Theatre, from the pen of Mr. Alfred Sutro—who has two other successful plays now running at West End theatres—appears likely to give entire satisfaction. "Mollentrave on Women" is a pleasant comedy, full of clever *mots* and provocative of much laughter. Mollentrave is considered, both by his friends and by himself, as an authority on women. He has studied the subject thoroughly, has been married three times so as to be well up in all details, and has written a book called "Mollentrave on Women" which has reached its 23rd edition, and which is looked upon as a dependable book of reference. The author is consulted by an eminent lawyer and politician who against his will is loved by his ward, and Mollentrave undertakes to put matters straight. His "putting straight" at first only succeeds in getting the wrong people engaged to each other, but ultimately the crumples in the rose-leaves are smoothed out, and the wisdom of Mollentrave is demonstrated. Mr. Eric Lewis and Miss Marion Terry are seen to great advantage in their respective parts.

### A Book and its Story.

MISS ETHEL McCAUL'S EXPERIENCES IN JAPAN.*

Miss Ethel McCaul left England in March last to fulfil the mission which had been entrusted to her of inspecting the work of the Red Cross Society in Japan. Queen Alexandra graciously gave her sanction and the Japanese Government its permission for the inspection. Miss McCaul was able to carry out her intention of becoming acquainted with the methods employed in military and civil hospitals, the organisation of the Army Nursing Service, and to visit the principal civil prisons. In the short time that she was in Japan she was able, by the courteous assistance rendered her by the War Office, in the matter of escorts and facilities for carrying out the inspection, to obtain an immense amount of information. This is given in a lucid and interesting manner in her book, which, aided by many photographs of persons and places, makes it a valuable addition to numerous others for which the Russian-Japanese War is indirectly responsible. It was naturally not without some misgivings that Miss McCaul set out on her expedition. She writes that during her long voyage doubts crossed her mind frequently as to whether she had been too presumptuous in undertaking such a mission. But subsequent events justified the belief that every facility would be given to carry out her plans. "I was aware that an enormous amount of priceless knowledge was to be gained from the Japanese on these subjects, and I was determined to gather as much information as possible, and through the courtesy of the authorities I most certainly did acquire what I was seeking." The secret of Miss McCaul's success lay probably in the tactful way in which she made use of the opportunities given, for from the outset she expressed to the authorities that the object of her mission was to seek instruction, and not to give it. To this she attributes the fact of her being allowed to see so much of the inner workings of the Japanese military and civil nursing institutions. "All I was privileged to see far exceeded my expectations; the Japanese are a marvellously clever people and their powers of organisation extraordinary. Amongst their most distinguishing traits are economy and exactness; an *economy* without stint and an *exactness* which involves the minutest attention to the smallest details."

Miss McCaul arrived at Tokio at the end of April, and two days later when anchored off Moji, she had her first view of a Japanese Red Cross Society's hospital ship. "She passed so close that we could discern her name—*Hakuai Maru* (The Benevolent Ship)—painted white, with a distinctive scarlet cross on her funnel. She was a fine, impressive-looking vessel and clearly recognisable as a hospital ship. The nurses were seen moving about in their white uniforms, and we expressed a hope that it might fall to our lot to be sent to Manchuria in one of these vessels." Miss McCaul afterwards went to the front in the *Hakuai Maru*, accompanied by Miss St. Aubyn, who had come out with her from England. They were joined by a Japanese lady, Madame Kuroda, who was sent by the War Office to represent it and act as interpreter. Madame Kuroda was an indispensable and indefatigable guide, to whom grateful acknowledgments are made. "She had a most intelligent face and spoke fluent English, but with a slightly American accent; her appearance and her dress were European." Self-control and reserve are cardinal virtues in Japan, as everyone knows. A speech at a luncheon party at Sir Claude Hamilton's soon after their arrival at Tokio is one of the many instances given as an illustration. The Japanese Minister of War and other distinguished members of Japanese official circles were present. "The Japanese were in very good spirits owing to the news having just reached them that Port Arthur had been successfully blocked. The modest spirit in which they receive the news of their brilliant successes, and the temperate way in which they speak of the Russians are quite remarkable. The following speech in quaint English, may illustrate more clearly my meaning. I had offered my congratulations on a recent victory: 'We hope we may be able to rejoice,' was the reply, 'but we must not do so over-much yet. We must hide our feelings and thoughts for the present; we are as children fighting grown-up children, our nation is still very young, and we are taught from our earliest childhood never to give way to excessive feelings.'" This lesson of self-control seems really to have been learnt by the nation and accounts for the very quiet and unemotional way in which the troops take their departure for the front; yet there is no lack of enthusiasm, and the spirit of patriotism is carried so far that the soldiers would rather die than be returned disabled. Devotion to their Emperor and country is a thing so sacred that no sacrifice is accounted too great, for they esteem it an honour and a privilege to die fighting. Surely this must help to explain the secret of their success against such a Power as Russia. Before leaving Tokio Miss McCaul was presented to the Empress. She was requested not to wear a hat, and to appear in a long light dress and wear her decorations." The Imperial Palace is a modern building and was rebuilt twelve years since. The spacious drawing-room is very beautiful, with doors and ceiling of finest Japanese workmanship. The hangings and furniture of the room are European and out of place in Oriental surroundings. The room in which the actual presentation took place was very simple and unpretentious; the Empress, who was standing, surrounded by her ladies in waiting, was most dignified, though very tiny, dressed in European fashion in mauve silk, with two large pearls in her hair; and a large pearl brooch and white kid gloves completed her toilet. The Chamberlain was the smallest of men, but he, like all the other officers of the Court, had most graceful and charming manners. The interview was a brief one; and after the presentation by Lady Macdonald the Empress shook hands with Miss McCaul and spoke in a very low voice, her lady-in-waiting interpreting, "her Majesty hoped the Queen of England was well; she was afraid I should have a very hard time at the front, but hoped to see me on my return."

The presentation was followed by a visit to the Red Cross Society's hospital. No one was able to speak English, and Madame Kuroda was kept busy interpreting. The doctors and nurses received the visitors with much ceremony. After the usual tea-drinking, they were conducted through the wards and theatre, having to remove their shoes before entering. They found everything scrupulously clean and in perfect order, but were struck with the bareness of the wards. There were no chairs or tables, the bedsteads had hard round pillows. But what to us are necessities are to the Japanese luxuries. Their standard of comfort is entirely different to our own. The nurses' home attached to the hospital is described as consisting of many clean little rooms with softly-padded matting floors. Small writing-tables, nine inches high, are the only furniture, except the lockers in which are kept the nurses' clothes, and as they passed through the rooms many who were "off duty" were sitting on the floor writing. We regret that space does not permit of further notice of this interesting and graphic account of Miss McCaul's experiences in Japan. Her book is one that everyone who is interested in the work of the Red Cross Hospital Society should read.

* Under the Care of the Japanese War Office. By Ethel McCaul. (Cassell and Co. 6s.)

### Notes and Queries.

**REGULATIONS.**

**The Editor is always willing to answer in this column, without any fee, all reasonable questions, as soon as possible.**

**But the following rules must be carefully observed :—**

**1. Every communication must be accompanied by the name and address of the writer.**

**2. The question must always bear upon nursing, directly or indirectly.**

**If an answer is required by letter a fee of half-a-crown must be enclosed with the note containing the inquiry.**

*Training in Birmingham.*

(155) Will you kindly tell me of a hospital in or near Birmingham for fever or general training, where they give a salary to probationers from the commencement of their duties ?—*A. D.*

See "The Nursing Profession: Where and How to Train," published by the Scientific Press.

*Roman Catholic.*

(156) Will you please let me know if it is possible for a Roman Catholic to get the position of district nurse in or near London ?—*S. A. S.*

There should be no difficulty.

*Massage.*

(157) Will you tell me the fees for learning massage, which is the best hospital, and how long is the shortest time one can get a certificate ?—*E. C.*

The fees vary, and the time for training for a certificate also varies. Write to the National Hospital for the Paralysed and Epileptic, Queen Square, W.C.; also to the Incorporated Society of Trained Masseuses, 12 Buckingham Street, Strand, W.C.

*Dispensary Training.*

(158) Will you kindly tell me where to get dispensary training in or near London ?—*Nurse R.*

Write to Miss M. E. Buchanan, M.P.S., Gordon Hall, Gordon Square, W.C.

*Paris.*

(159) In your paper a short time back there was an advertisement for the ——— Hospital, for a nurse, which a friend of mine answered. I should esteem it a great favour if you would kindly let me know at your earliest convenience some particulars as to the respectability of same, as she is a young lady having no friends in Paris.—*H. M. R.*

We know of nothing whatever against the hospital.

*Army Nurses.*

(160) Will you kindly give me information as to how to become an army nurse, and where to apply for the necessary information ? —*B. G. D.*

If you desire to join Queen Alexandra's Imperial Military Nursing Service write to the Under Secretary of State, War Office, Pall Mall, S.W.

*Home.*

(161) Can you recommend an inexpensive home for elderly gentleman, feeble-minded and eccentric, or a charitable institution where he would be received for a small payment for permanency ? —*E. B.*

There is a full list in "Burdett's Hospitals and Charities," published by the Scientific Press, 28 and 29 Southampton Street, Strand, W.C.

*Registration of Midwives.*

(162) Will you please inform me if the law of registration of midwives applies to Scotland, or is it just England ? And if it is applied to Scotland where would I get registered ? I have been a monthly nurse for several years, but I only got trained and received my certificate for midwifery last June.—*Anxious.*

This Act applies to midwives in England and Wales, but not to Scotland. Any further details may be obtained from "How to Become a Midwife," published by the Scientific Press, 28 and 29 Southampton Street Strand, W.C.

**Handbooks for Nurses.**

| | Post Free. |
|---|---|
| How to Become a Nurse: "The Nursing Profession" ... | 2s. 4d. |
| "Nurses' Pronouncing Dictionary." Terms and Treatment... ... ... ... ... ... ... ... | 2s. 0d. |
| "A Handbook for Nurses." Dr J. K. Watson ... ... | 5s. 4d. |
| "Elementary Physiology for Nurses" ... ... ... | 2s. 0d. |
| "Swedish Massage and Movements" ... ... ... | 2s. 6d. |

Of all booksellers or of The Scientific Press, Limited, 28 & 29 Southampton Street, Strand, London, W.C.

### For Reading to the Sick.

WEARY IN WELL DOING.

I WOULD have gone ; God bade me stay ;
I would have worked ; God bade me rest.
He broke my will from day to day,
He read my yearnings unexpressed
And said them nay.

Now I would stay ; God bids me go ;
Now I would rest ; God bids me work.
He breaks my heart tossed to and fro.
My soul is wrung with doubts that lurk
And vex it so.

I go, Lord, where Thou sendest me ;
Day after day I plod and moil ;
But Christ, my God, when will it be
That I may let alone my toil
And rest with Thee ?

*Christina Rossetti.*

"He ever liveth to make intercession for them."—*Heb.* vii. 25.

It is a meditation fraught with comfort to believe that our Lord, enthroned in glory at the Father's right hand, while receiving the homage of the countless hosts of the unfallen creation, has still His interests in the Church on earth ; that not only does He know what is going on in each individual human soul, but that He responds to its wants and aspirations, that He sympathises with its sorrows, and concerns Himself in its hopes and fears. Nay more, most satisfying is the feeling that, with delicate thought and nice discrimination, He applies to each one of us just that medicament which the sick and weary soul stands in need of. He knows exactly what grace to supply—at once the good physician and the Cure.—*Bishop Forbes.*

A few struggles at most, few in proportion to the joy set before us, and the last struggle will resign us over to the end of our struggles, even endless peace, of which the peace vouchsafed to each faithful struggle is the earnest and the preparation : a few years past, and what will it concern us—under what circumstances we have passed our life, so that our lot is then with God's Saints ? What will matter, then—privation, sorrow, disappointment, dejection of heart, failing of the eyes, suffering of the body ? Yea, rather, as our loving Lord spoke, how blessed it will have been to have "mourned" if by His mercy we may then "be comforted" ; how blessed to have been "poor in spirit," if then through His merits ours be "the Kingdom of Heaven" ; how blessed any self-denial, or toil, or pain, or chastisement, whereby purity of heart shall have been retained or restored, if, when we shall close our eyes upon the vanities and distractions of this passing world, and open them upon eternity, we shall "see God !" Which may He of His infinite mercy grant, not for our worthiness, but to our unworthiness, for the worthiness of His ever-blessed Son, in Whom He "hath made" such as we are, and can make us also "to be meet to be partakers of the inheritance of the Saints in light."

*E. B. Pusey.*

God doth not bid thee wait,
To disappoint at last ;
A golden promise fair and great
In precept-mould is cast.
Soon shall the morning gild
The dark horizon-rim,
Thy heart's desire shall be fulfilled,
Wait patiently for Him.

*F. R. Havergal.*

